# CHK1 regulates NF-κB signaling upon DNA damage in p53- deficient cells and associated tumor-derived microvesicles

**DOI:** 10.18632/oncotarget.7566

**Published:** 2016-02-22

**Authors:** Brittany L. Carroll, Michael J. Pulkoski-Gross, Yusuf A. Hannun, Lina M. Obeid

**Affiliations:** ^1^ Stony Brook Cancer Center and The Department of Medicine, Stony Brook, New York, USA; ^2^ Pharmacological Sciences, Stony Brook University, Health Sciences Center, Stony Brook, New York, USA; ^3^ Northport Veterans Affairs Medical Center, Northport, New York, USA

**Keywords:** p53, CHK1, DNA damage, tumor microvesicles

## Abstract

The recently discovered CHK1-Suppressed (CS) pathway is activated by inhibition or loss of the checkpoint kinase CHK1, promoting an apoptotic response to DNA damage mediated by caspase-2 in p53-deficient cells. Although functions of the CS-pathway have been investigated biochemically, it remains unclear whether and how CHK1 inhibition can be regulated endogenously and whether this constitutes a key component of the DNA damage response (DDR). Here, we present data that define the first endogenous activation of the CS-pathway whereby, upon DNA damage, wild type p53 acts as an endogenous regulator of CHK1 levels that modulates caspase-2 activation. Moreover, we demonstrate that persistence of CHK1 levels in response to DNA damage in p53-deficient cancer cells, leads to CHK1-mediated activation of NF-κB and induction of NF-κB-regulated genes in cells and in associated tumor-derived microvesicles (TMVs), both of which are abrogated by loss or inhibition of CHK1. These data define a novel role for CHK1 in the DDR pathway as a regulator NF-κB activity. Our data provide evidence that targeting CHK1 in p53-deficient cancers may abrogate NF-κB signaling that is associated with increased cellular survival and chemoresistance.

## INTRODUCTION

Alterations in p53 are amongst the most frequent in all human cancers and are associated with increased capacity to evade apoptosis leading to therapeutic resistance; consequently elucidating p53-independent DDR pathways that could overcome this resistance has been an intense area of research [1-4]. Recently the CS-pathway has emerged as a novel apoptotic pathway [4, 5]. CHK1 is a serine/threonine kinase and an effector of the DDR that, once activated, acts to halt progression through the cell cycle by phosphorylating downstream targets [6]. Importantly, during the CS-pathway, loss or inhibition of CHK1 in the context of mutant p53 circumvents p53 status to promote a caspase-2 apoptotic response to DNA damage [4, 5].

Caspase-2 is a poorly defined but evolutionarily conserved member of the caspase family. It is activated by proximity-induced oligomerization via recruitment to a high molecular weight platform termed the PIDDosome. The PIDDosome is comprised of three proteins, PIDD (p53-induced death domain protein) and RAIDD (RIP associated Ich-1/CED homologous protein with death domain) that interact through death domains existing in both proteins. Caspase-2 is recruited to RAIDD via the caspase recruitment domain present in both proteins [7]. Interestingly, in addition to activating caspase-2, PIDD can also activate NF-*κ*B in response to DNA damage forming a separate PIDDosome complex that lacks RAIDD and caspase-2 but contains RIPK1 and NEMO (NF-*κ*B essential modulator)/IKK*γ* [8]. Unlike caspase-2, NF-*κ*B is a transcription factor whose activity results in induction of pro-survival genes in addition to a number of chemokines and cytokines [9]; therefore, PIDD can elicit either pro-survival or pro-death signals according to its protein interactions [10].

The DDR is a complex and interconnected signaling network that controls cell fate; therefore the proteins in this network must be tightly regulated. Consequently, the relationship between CHK1 and p53, both important proteins in the DDR, is complex. CHK1 can phosphorylate p53, which is important for p53 stabilization in response to DNA damage [11]; on the other hand, p53 can induce CHK1 down-regulation although the functional significance has not been determined [12, 13]. Moreover, high CHK1 expression positively correlates with tumor grade and disease recurrence and is associated with therapeutic resistance [14-17]. The presence of two distinct PIDDosome complexes that elicit opposing effects within the cell and whose assembly may be differentially regulated by CHK1 levels and potentially p53 adds to the complexity and importance of deciphering this signaling pathway. Furthermore, although CHK1 has been identified as an inhibitor of caspase-2 PIDDosome formation, its effect on NF-*κ*B activation is unknown and could have great clinical significance. Given the importance of these pathways on cell fate and the intricacies of the signaling network, this study aims to investigate the role of p53 in the CS-pathway and identify the effects perturbations of this pathway have on caspase-2 and NF-*κ*B signaling that may ultimately dictate cell fate and chemotherapeutic response.

## RESULTS

### Regulation of CS-pathway by DDR and role of P53

**Figure 1:**
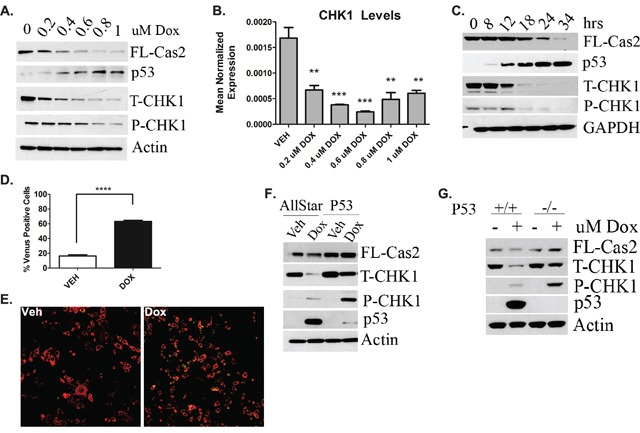
Regulation of CS-pathway by DDR and Role of P53 MCF7 cells were treated with the indicated dose of doxorubicin for 24h. Cells were then **A.** harvested in RIPA buffer and total cell lysate was analyzed by western blot for the proteins indicated or **B.** harvested in RLT buffer, prepared for quantitative reverse transcriptase-PCR, with enzyme expression normalized to b-actin expression for each reaction in triplicate. Data are presented as mean ± SEM of 3 independent experiments. **C.** MCF7 cells were treated with 0.8uM doxorubicin for the indicated times, harvested and total cell lysate was analyzed by western blot for the proteins indicated. **D.** MCF7 cells were transiently transfected with C2-CARD VN (500ng) and C2-CARD VC (500ng) along with pshooter.dsRed-mito (250ng) as a reporter for transfection. Twenty-four hours after transfection, cells were treated with 0.8uM doxorubicin for 24h and then the percentage of pshooter.ds.Red-mito-positive (red) cells that were Venus positive (green) was determined from a minimum of 100 cells per plate. Data are presented as mean ± SEM of 3 independent experiments. **E.** Representative confocal images of cells from (D) are shown. **F.** MCF7 cells were transfected with p53 siRNA (20nM). Forty-eight hours after transfection, cells were treated with 0.8uM doxorubicin for 24 hours, harvested and total cell lysate was analyzed by western blot for the proteins indicated. **G.** HCT-116 colon cancer cells either wild type (+/+) or null (−/−) for p53 were treated with 0.8uM doxorubicin for 24 hours, harvested in RIPA buffer and total cell lysate was analyzed by western blot for the proteins indicated.

Although the functions of the CS-pathway have been probed through pharmacological inhibition and siRNA knockdown [4, 5], it is unclear whether and how CHK1 inhibition can be regulated endogenously. In initial studies we set out to determine effects of the DDR on CHK1 and caspase-2. To this end, levels of p53 and CHK1 were assessed as well as caspase-2 processing in response to doxorubicin in MCF7 breast cancer cells that are wild type (WT) for p53. Doxorubicin dose-dependently resulted in p53 accumulation, concomitant with CHK1 down-regulation at the protein (Figure 1A) and message level (Figure 1B). This was also accompanied by caspase-2 processing. Next to determine kinetics of p53 accumulation, CHK1 down-regulation, and caspase-2 activation, a time course assay was performed using 0.8uM doxorubicin, a dose in which CHK1 levels were approximately 80% reduced at the protein level and there was significant reduction in pro-caspase-2 (Figure 1A). Consistent with the dose response, p53 accumulation was concomitant with a significant down-regulation of CHK1 followed by caspase-2 processing (Figure 1C). Caspase-2 activation was also observed in approximately 60% of total cells at 24 hours, as measured by Bimolecular Fluorescence Complementation (BIFC) (Figure 1D). Representative BIFC confocal images are shown in Figure 1E. Taken together, these results demonstrate that the DDR is sufficient to induce loss of CHK1, thus identifying stimulus regulation of CHK1 levels.

Next, to determine whether the observed CHK1 down-regulation was p53-mediated, siRNA was used to deplete p53 to assess the effects on CHK1 levels and caspase-2 processing. Indeed p53 knockdown rescued doxorubicin-induced CHK1 down-regulation and also restored levels of pro-caspase-2 (Figure 1F). To corroborate these data, the p53 isogeneic colon cancer cell lines HCT-116 either WT (+/+) or null (−/−) for p53 were investigated. The HCT-116 p53+/+ cells showed down-regulation of CHK1 and caspase-2 processing upon doxorubicin treatment that was abrogated in the HCT-116 p53−/− cells, similar to p53 knockdown in MCF7 cells (Figure 1G).

Collectively, these data provide evidence that the DDR and p53 regulate CHK1 and caspase-2 activation, defining the first mechanism of endogenous activation of the CS-pathway.

### The CS-pathway can be activated in wild type p53 cells

**Figure 2:**
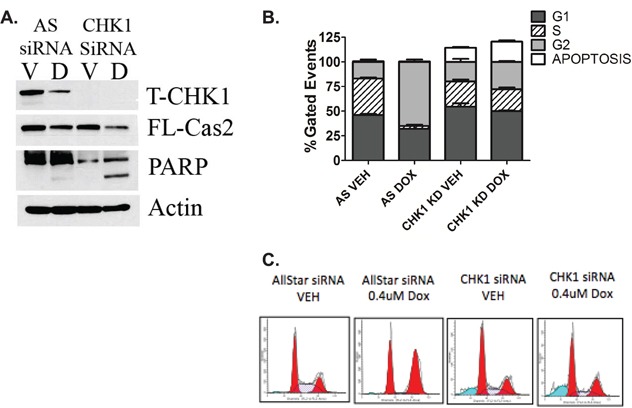
The CS-pathway can be activated in wild type p53 cells MCF7 cells were transfected with CHK1 siRNA (20nM). Forty-eight hours after transfection, cells were treated with 0.4uM doxorubicin for 24 hours and either harvested in RIPA and total cell lysate analyzed by western blot for the proteins indicated **A.** or harvested, fixed and labeled with propidium iodide for cell cycle analysis **B.** Data are presented as mean ± SEM of 3 independent experiments. **C.** Images of cell cycle analyzed by ModFit LT software.

The CS-pathway has been traditionally investigated in the context of mutant p53, therefore we wanted to investigate whether the CS-pathway could be activated in WT p53 cells at lower doses of doxorubicin where CHK1 levels remain high (Figure 1A) To determine whether abrogation of CHK1 signaling is sufficient to activate the CS-pathway in WT p53 cells, two different methods were employed: depletion of CHK1 by siRNA (Figure 2A) and inhibition of CHK1/2 activity through the use of the ATP competitive inhibitor AZD-7762 (Fig S1A) in combination with 0.4uM doxorubicin, a low dose where CHK1 levels remain detectable and caspase-2 processing is minimal (Figure 1A). The results showed that in the absence of CHK1 there was an approximately 50% reduction in pro-caspase-2 upon doxorubicin treatment at 0.4uM concentration compared to doxorubicin alone (Figure 2A). Similarly under the same conditions, inhibition of CHK1 activity resulted in a decrease in pro-caspase-2 (Fig S1A) as well as a 2-fold increase in the number of Venus-positive cells with CHK1 inhibition in combination with 0.4uM doxorubicin compared to doxorubicin alone as measured by BIFC microscopy (Fig S1B). Also of note, CHK1 knockdown resulted in the abrogation of doxorubicin-induced G2/M arrest and a significant increase in the amount of apoptotic cells (Figure 2B and 2C), suggesting that apoptosis can be achieved at a lower dose of doxorubicin in combination with inhibition/loss of CHK1 in WT p53 cells.

Altogether these data suggest that the CS-pathway can be activated in WT p53 cells in response to DDR, to initiate apoptosis as opposed to cell cycle arrest, which has been associated with drug resistance [18].

### P53 deficiency triggers deregulation of the CHK1-caspase-2 pathway

It was recently demonstrated that targeting CHK1 is therapeutically beneficial in p53 deficient cells as well as in triple negative breast cancers (TNBC), which harbor alterations in p53 at a frequency of approximately 40% of cases (14, 19, 20). Given the clinical relevance of targeting CHK1 in p53-mediated cancers and our data demonstrating WT p53 regulates CHK1 and the CS-pathway, it became important to investigate whether p53 deficiency promotes deregulation of the CHK1-caspase-2 pathway.

**Figure 3:**
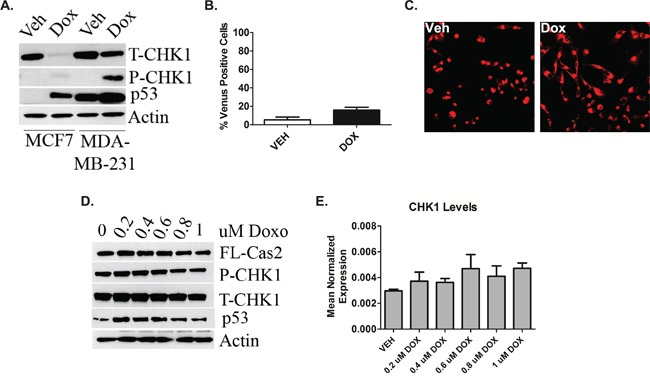
P53 deficiency triggers deregulation of the CHK1-caspase-2 pathway **A.** MCF7 and MDA-MB-231 cells were treated 0.8uM doxorubicin for 24 hours, harvested and total cell lysate was analyzed by western blot for the proteins indicated. **B.** MDA-MB-231 cells were transiently transfected with C2-CARD VN (500ng) and C2-CARD VC (500ng) along with pshooter.dsRed-mito (250ng) as a reporter for transfection. Twenty-four hours after transfection, cells were treated with 0.8uM doxorubicin for 24 hours and then the percentage of pshooter.ds.Red-mito-positive (red) cells that were Venus positive (green) was determined from a minimum of 100 cells per plate with representative confocal images of cells from **(B)** shown **(C)**. Data are presented as mean ± SEM of 3 independent experiments. MDA-MB-231 cells were treated with the indicated dose of doxorubicin for 24 hours. Cells were then harvested in RIPA buffer and total cell lysate was analyzed by western blot for the proteins indicated **D.** or harvested in RLT buffer, prepared for quantitative reverse transcriptase-PCR, with enzyme expression normalized to b-actin expression for each reaction in triplicate **E.** Data are presented as mean ± SEM of 3 independent experiments.

Experiments were conducted in the TNBC cell line MDA-MB-231 that also harbors a missense mutation R280K in p53. In contrast to MCF7 cells, doxorubicin treatment did not result in down-regulation of CHK1 (Figure 3A). Next to determine whether doxorubicin-induced caspase-2 activation occurs in MDA-MB-231 cells BIFC microscopy was utilized. MDA-MB-231 cells showed no significant increase in caspase-2 dimerization post-doxorubicin treatment compared to vehicle treated cells (Figure 3B). Representative images are shown in Figure 3C. Similarly, a doxorubicin dose response was also performed. Down- regulation of CHK1 was not detected at any dose at the protein (Figure 3D) or message (Figure 3E) level nor was decrease in pro-caspase-2 (Figure 3D).

Next, to determine whether deregulation of the CHK1-caspase-2 pathway occurred in other cell lines with p53 deficiency, humanized mutant p53 knock-in (HUPKI) mouse embryonic fibroblasts (MEFs) harboring the G245S hotspot mutation were treated with doxorubicin. Similar to MCF7 cells, WT MEFs showed significant down-regulation of CHK1 and processing of pro-caspase-2 in response to doxorubicin that was abrogated in G245S MEFs (Fig S2).

Collectively, these data establish that p53 deficiency promotes sustained cellular CHK1 levels in response to genotoxic stress that inhibits caspase-2 activation whereas p53 sufficiency can launch the CS-caspase-2 pathway upon induction of the DDR.

### CHK1 levels regulate NF-κB signaling in p53-deficient cells in response to doxorubicin

**Figure 4:**
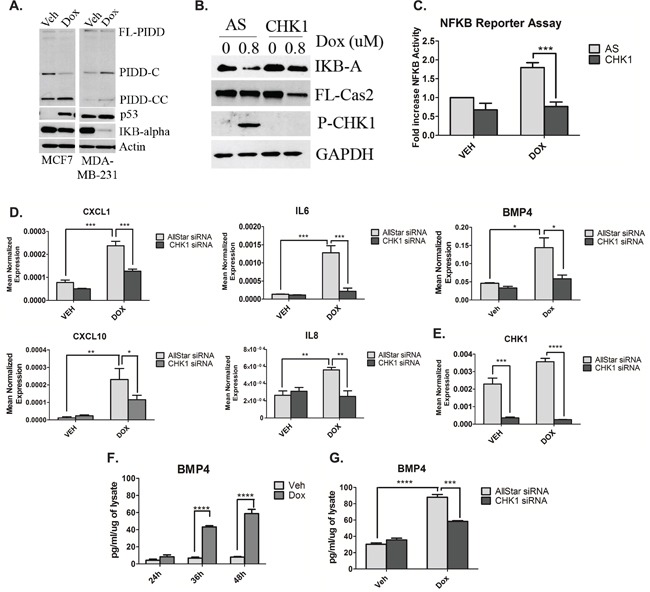
CHK1 levels regulate NF-κB signaling in p53-deficient cells in response to doxorubicin **A.** MCF7 and MDA-MB-231 cells were treated 0.8uM doxorubicin for 24 hours, harvested and total cell lysate was analyzed by western blot for the proteins indicated. **B.** MDA-MB-231 cells were transfected with CHK1 siRNA (20nM). Forty-eight hours after transfection, cells were treated with 0.8uM doxorubicin for 24 hours, harvested and total cell lysate was analyzed by western blot for the proteins indicated. **C.** MDA-MB-231 cells were co-transfected with 1ug of NFKB promoter-luciferase construct and V5 luciferase construct for 18 hours followed by treatment with 0.8uM doxorubicin for 24 hours. Luciferase and galactosidase activities were extracted and assayed as described in “Material and Methods” and measured luciferase activity was normalized to measured galactosidase activity. Data are presented as mean ± SEM of 3 independent experiments. **D** and **E.** MDA-MB-231 cells were transfected with CHK1 siRNA (20nM). Forty-eight hours after transfection, cells were treated with 0.8uM doxorubicin for 24 hours, harvested in RLT buffer and prepared for quantitative reverse transcriptase-PCR, with enzyme expression normalized to b-actin expression for each reaction in triplicate. Data are presented as mean ± SEM of 3 independent experiments. **F.** MDA-MB-231 cells were treated with 0.8uM doxorubicin for the indicated times, media was then collected and ELISA was performed to assess levels of protein in the media. **G.** The media from (D) was then used for ELISA. Data are presented as mean ± SEM of 3 independent experiments.

As mentioned previously, two distinct PIDDosome signaling complexes exist that exert opposing effects within the cell; one resulting in caspase-2 activation and a second that activates pro-survival NF-κB signaling. In light of the results from Figure 3 demonstrating that p53 deficient cells display high levels of CHK1, that inhibits caspase-2 activation, we suspected that doxorubicin may paradoxically (and counterproductively) cause activation of NF-κB via the alternate PIDDosome. To this end, experiments were performed to determine whether doxorubicin induced degradation of IKB-α, an endogenous inhibitor of NF-κB translocation to the nucleus and subsequently NF-κB activity. Indeed, upon doxorubicin treatment, IKB-α became almost undetectable in MDA-MB-231 cells, reduced at the protein level by approximately 95%, while MCF7 cells showed no significant reduction in IKB-α levels (Figure 4A). Interestingly, PIDD auto-processing also differed between these two cells lines. PIDD-C, the fragment associated with NF-κB activation was the predominant fragment in MDA-MB-231 cells upon doxorubicin treatment, while the fragment associated with caspase-2 activation, PIDD-CC, was the most abundant fragment in MCF7 cells after treatment with doxorubicin (Figure 4A). Given the pro-survival signaling associated with NF-κB activity, it became important to determine whether CHK1 knockdown could abrogate IKB-α degradation and therefore inhibit NF-κB activity. As shown in Figure 4B, loss of CHK1 abolished IKB-α degradation and induced caspase-2 processing in MDA-MB-231 cells. To corroborate these data in a functional manner, NF-κB promoter activity was measured. An approximate 1.8-fold increase in NF-κB promoter activity was observed upon doxorubicin treatment that was abrogated by CHK1 knockdown in MDA-MB-231 cells (Figure 4C). Similar results were also observed in the HUPKI G245S MEFs using the CHK1/2 inhibitor AZD7762. (Fig S3A). In accordance with the data from Figure 4A, no induction in NF-κB promoter activity was observed in MCF7 upon doxorubicin treatment (Fig S3B). These results demonstrate activation of NF-κB by doxorubicin in p53-deficient cells, and suggest that loss or inhibition of CHK1 could abrogate doxorubicin-induced NF-κB signaling in mutant p53 cells.

To determine the biological significance of doxorubicin-induced NF-κB signaling and the effect of CHK1 knockdown, levels of chemokines and cytokines of which many are known targets of NF-κB were evaluated by utilizing a quantitative PCR array. Of the 84 genes examined, knockdown of CHK1 in the presence of doxorubicin showed down-regulation of 81% of the genes, with 20% of these genes showing significant down-regulation greater than 50% when compared to doxorubicin alone (Fig S3C). Moreover, real-time PCR analysis using independent primers of several genes was employed to corroborate the PCR array results. Loss of CHK1 significantly reduced the doxorubicin-mediated induction of the CXC chemokines CXCL1, CXCL10, IL8 (Interleukin-8) and the cytokines IL-6 (Interleukin-6) and BMP4 (Bone Morphogenetic Protein 4) (a member of the TGF-B family) at the mRNA level (Figure 4D). Significant knockdown of CHK1 at the message level was achieved (Figure 4E). To confirm the changes in mRNA after doxorubicin treatment were reflected in secreted protein levels, an ELISA for both BMP4 and IL6 was performed. Significant increases in secreted BMP4 protein was observed at 36 hours after doxorubicin, increasing over time to an approximately 4-fold increase at 48 hours (Figure 4F). Similarly, a 3-fold increase in secreted IL6 protein was observed at 24 hours post-doxorubicin treatment (Fig S3D). Next, to confirm CHK1 knockdown resulted in a reduction in secreted BMP4 protein, a subsequent ELISA was performed. Indeed, CHK1 knockdown in combination with doxorubicin treatment resulted in a significant reduction in secreted BMP4 compared to doxorubicin treatment alone at 36 hours (Figure 4G).

Altogether, these results demonstrate robust effects of doxorubicin on NF-κB and NF-κB-regulated cytokines and chemokines. Furthermore, the results define a novel role for CHK1 in regulating the NF-κB response of p53-deficient cells to the doxorubicin.

### Doxorubicin induces the shedding of TMVs containing NF-κB-responsive genes

Communication between cells mainly involves the secretion of soluble proteins, such as BMP4 and IL6, that interact and elicit signaling responses through binding of receptors on neighboring cells [21]; however another form of cellular communication has emerged centered on the release of membrane microvesicles [22, 23]. Research has shown that TMVs are shed from very invasive and aggressive cancer cells such as MDA-MB-231 cells and these membrane-bound vesicles contain molecular information that can be transferred to recipient cells and can greatly affect the tumor microenvironment [24, 25]. Interestingly during confocal experiments, pronounced vesicle formation was observed upon doxorubicin treatment. Therefore, it became important to determine whether MDA-MB-231 cells shed TMVs and whether these TMVs contain inflammatory mediators in response to doxorubicin.

**Figure 5:**
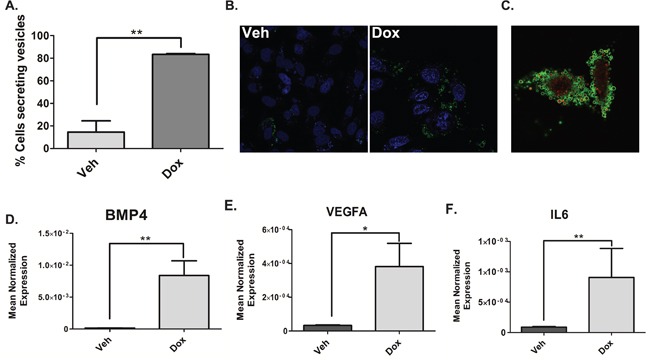
Doxorubicin induces the shedding of TMVs containing NF-κB-responsive genes **A.** MDA-MB-231 cells were treated 0.8uM doxorubicin for 68 hours. After 68 hours cells were stained with DRAQ5 to label all cells followed by labeling with Annexin-V and Propidium to specifically label TMVs and live cell imaging was performed. The number of cells secreting TMVs out of the total number of cells in the field was counted and quantified, blindly. Data are presented as mean ± SEM of 3 independent experiments. **B.** MDA-MB-231 cells were treated 0.8uM doxorubicin for 68 hours. After 68 hours Annexin-V and Propidium were added to the media to label TMVs. Live cell imaging was then performed on the samples (B) is a representative image. **(C-F)** MDA-MB-231 cells were treated with 0.8uM doxorubicin for 68 hours. Cells were harvested in RIPA buffer. The media was collected and subjected to differential centrifugation (outlined in “material and methods”). RNA was then extracted from the 50,000xG pellet and the cells and prepared for quantitative reverse transcriptase-PCR, with enzyme expression normalized to b-actin expression for each reaction in triplicate. Data are presented as mean ± SEM of 3 independent experiments.

To this end confocal microscopy was performed and annexin-V was used to detect TMVs as the membranes of these vesicles contain phosphatidylserine. Propidium iodide was used to label nucleic acids within the TMVs. Basally, approximately 20% of vehicle-treated cells were observed to shed TMVs while doxorubicin induced the shedding of microvesicles in approximately 80% of the total number of cells counted (Figure 5A). Representative images are shown in Figure 5B. Figure 5C shows significant shedding of TMVs containing nucleic acids upon doxorubicin treatment. A time-lapse video showing recruitment of TMVs to surrounding cells is shown in Figure S4A. Next, to examine the mRNA cargo inside these vesicles, differential centrifugation was used to isolate TMVs found within the 50,000xg pellet followed by RNA extraction and real-time PCR. Interestingly, the mRNA of a number of genes including IL6 (Figure 5D), BMP4 (Figure 5E) and VEGF (Figure 5F) were significantly elevated in the TMVs from the doxorubicin treated cells compared to vehicle. To ensure the population of TMVs isolated was pure, a marker of TMVs, Arf6, was utilized. Western blot analysis showed the presence of Arf6 exclusively in the 50,000xg pellet and enriched in the doxorubicin sample while it was absent in the 100,000xg exosome-containing pellet (Fig S4B).

Altogether these data establish doxorubicin-induced TMV shedding in MDA-MB-231. Furthermore, the TMVs similar to the cells contain elevated levels of chemokines and cytokines that can not only affect the tumor microenvironment, but also travel long distances to affect cells in distant sites, such as sites of metastasis.

### Loss of CHK1 alters TMV cargo

**Figure 6:**
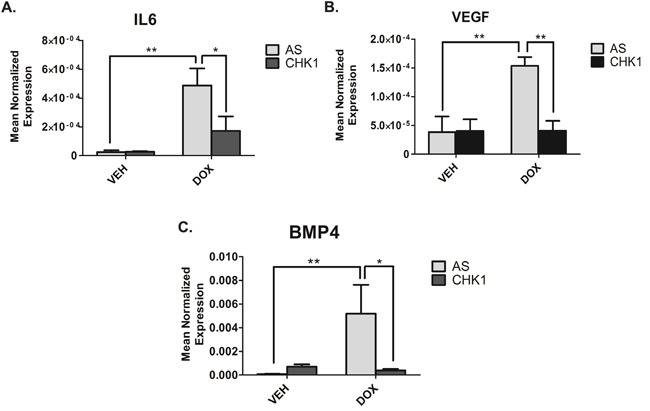
Loss of CHK1 alters the cargo of TMVs **A-C.** MDA-MB-231 cells were transfected with CHK1 siRNA (20nM). Eighteen hours after transfection, cells were treated with 0.8uM doxorubicin for 68 hours. Cells were harvested in RIPA buffer. The media was collected and subjected to differential centrifugation (outlined in “material and methods”). RNA was then extracted from the 50,000xG pellet and the cells and prepared for quantitative reverse transcriptase-PCR, with enzyme expression normalized to b-actin expression for each reaction in triplicate. Data are presented as mean ± SEM of 3 independent experiments.

We next sought to investigate the role of CHK1 in regulating TMVs and/or their cargo. Initial studies showed that CHK1 knockdown did not affect the number of TMVs secreted (data not shown). Subsequently, real-time PCR analysis of the 50,000xg pellet, showed loss of CHK1 significantly reduced the doxorubicin-mediated induction of IL6 (Figure 6A), VEGF (Figure 6B) and BMP4 (Figure 6C) in isolated TMVs compared to doxorubicin alone. Collectively, these data establish that CHK1 can modulate TMV cargo in response to doxorubicin.

## DISCUSSION

The results from this study provide new insights into how the DDR and WT p53 regulate CHK1, providing a previously unknown function for p53-mediated down-regulation of CHK1 in activating the CS-pathway. Moreover we investigated the deleterious effects on this regulatory network when p53 is altered and found aberrant NF-κB signaling that has been linked to many malignant diseases and chemotherapeutic resistance in p53-deficient cells could be abrogated through targeting CHK1.

**Figure 7:**
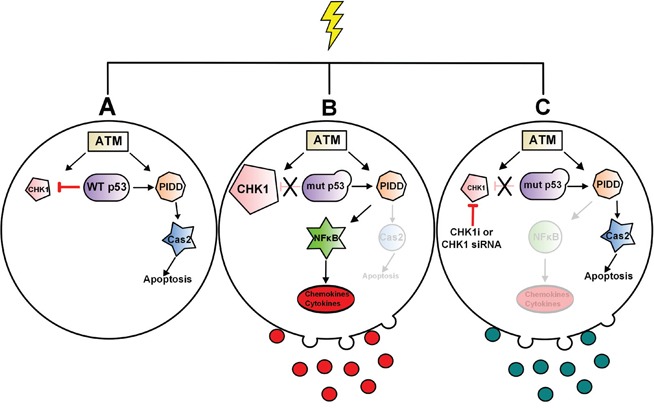
Model of p53 regulation of CS-pathway and CHK1 regulation NF-κB signaling in p53-deficient cells and associated TMVs In response to high dose of doxorubicin WT p53 is required to downregulate CHK1 in order to activate the CS-pathway and Caspase-2 **A.** In p53-deficient cells, p53 can no longer downregulate CHK1 resulting in high cellular levels, CHK1-mediated activation of NF-κB and an increase in NF-κB-responsive genes both in the cell and in associated-TMVs **B.** Loss or inhibition of CHK1 abrogates NF-κB signaling in response to doxorubicin **C.**

This work identified the first stimulus (doxorubicin) of the CS-pathway and established that WT p53 acts as an endogenous inhibitor of CHK1 upon DNA damage to activate the CS-pathway. In p53-deficient cells, we demonstrate a novel role for CHK1 in regulating NF-κB activity and the production of several pro-survival and pro-metastatic genes within the cell and also within TMVs in response to genotoxic stress (Figure 7). The significance of these findings is underscored by the fact that CHK1 has already been identified as an important pharmacological target. Importantly, thus far two possible mechanisms have been proposed to explain CHK1 inhibitor sensitivity: increased oncogenic replicative stress and reduced DNA repair capabilities, both of which are associated with CHK1's role in the cell cycle. Our work identifies a potential novel mechanism for CHK1 inhibitor sensitivity, through regulation of pro-survival NF-κB signaling.

P53 mediates many effects through activation of genes that regulate cell cycle checkpoints, DNA damage and repair, and apoptosis. Interestingly, two reports have demonstrated p53-mediated down-regulation of CHK1 [12, 13]. Although these studies only speculate as to why p53 has evolved to down-regulate CHK1, reports have correlated disruption of this regulatory pathway with poor prognosis and decreased survival of breast cancer patients [13]. Our study defines a previously unidentified role of p53-mediated down-regulation of CHK1 in activating the CS-pathway leading to caspase-2 activation.

As p53 is altered in over half of all human cancers [26], we set out to determine whether alterations of p53 promote deregulation of the CHK1-caspase-2 pathway. We demonstrate that p53-deficiency promotes sustained CHK1 levels in the presence of genotoxic stress. In accordance, with previous studies [4, 5] we show that high CHK1 levels inhibit the CS-pathway and caspase-2 activation but importantly, we also establish a novel function of CHK1 in regulating NF-κB activity and induction of known pro-survival and pro-metastatic mediators such as IL6, VEGF and BMP4 in p53-deficient cells upon doxorubicin treatment. Of note several recent studies have shown that p53-deficiency promotes NF-κB signaling [27, 28]. Specifically, it was demonstrated that mutant p53 via gain-of-function activity prolongs NF-κB activation and promotes chronic inflammation and inflammation-associated colorectal cancer *in vivo* [27]. Similarly, a second study showed that p53 deficiency is necessary for doxorubicin induced transcriptional activation of NF-кB target genes associated with invasion in human breast cancer and this was correlated with reduced disease free-survival of breast cancer patients [28]. In light of these data, the authors hypothesized that targeting NF-кB in p53-deficient cancers that respond to chemotherapeutics by activating NF-кB could be therapeutically beneficial. Our work in unraveling the mechanism that drives p53-deficient cells to activate NF-кB in response to doxorubicin provides evidence that inhibiting CHK1 in these cancers maybe a better alternative, as targeting transcription factors has proven challenging [29].

Interestingly, we also found that doxorubicin treatment of MDA-MB-231 cells resulted in a significant increase in the amount of shed TMVs and the enrichment of a number of chemokines, cytokines and growth factors inside these vesicles. TMVs are carriers of molecular information that act as signaling platforms, diffusing into the extracellular space to target cells in the microenvironment, modulate the interactions of tumor cells and also prime the formation of the metastatic niche [22, 25]. The packaging of mRNA of chemokines, cytokines and growth factors inside TMVs provides another means of cell-to-cell communication outside of conical secretory pathways that can greatly influence the tumor microenvironment. We found that although loss of CHK1 does not affect the amount of vesicles shed, it does modulate the cargo within the vesicles, significantly reducing the mRNA levels of a number of genes associated with survival and metastasis compared to doxorubicin treatment alone.

TMVs have recently gained attention as potential biomarkers as tumor cells release these vesicles into body fluids such as urine, blood and saliva where they can then be isolated and analyzed [22, 30]. Interestingly, our work demonstrates that TMVs become enriched with NF-кB target genes in response to doxorubicin in p53-deficient cells, mirroring what occurs inside the cell. This is important because as mentioned previously, several studies have shown poor therapeutic outcome in cancers that activate NF-кB in response to chemotherapeutics [28, 31]. These data provide evidence that isolating TMVs from body fluids may provide a rapid, noninvasive and economical way to monitor therapeutic efficacy specifically in cancers where repeated biopsies are not feasible and allow for early modulation of therapeutic regime.

In conclusion, our results establish a novel benefit of CHK1 inhibition, outside of promoting “mitotic catastrophe,” in the inhibition of NF-κB signaling in response to genotoxic stress in p53 deficient cells; thus providing more evidence in support of discovering new more specific CHK1 inhibitors. Although this work begins to establish CHK1 as a critical downstream target of p53 tumor suppressor activity and to unravel the multiple signaling contexts outside of the cell cycle that are affected by CHK1 inhibition further studies are needed to fully elucidate this signaling network and are essential for successful therapeutic development of CHK1 inhibitors.

## MATERIALS AND METHODS

### Materials

Lipofectamine® RNAiMAX, Annexin-V and Propidium Iodide were purchased from Life Technologies (Grand Island, NY), X-tremeGENE 9 DNA Transfection Reagent from Roche Diagnostics (Indianapolis, IN), iTAQ and SYBR® Green master mix master mix from Bio-Rad (Hercules, CA), and CHK1/2 inhibitor AZD7762 from Selleckchem (Houston, TX).

### Cell culture

MCF7 cells were purchased from ATCC and cultured in RPMI 1640 medium with 10% fetal bovine serum (FBS) both from Life Technologies (Grand Island, NY), MDA-MB-231 and HCT116 p53+/+ and HCT116 p53−/− from ATCC and cultured in Dulbecco's modified Eagle's medium (DMEM) with 10% FBS both from Life Technologies (Grand Island, NY). Humanized mutant p53 knock-in (HUPKI) mouse embryonic fibroblasts (MEFs) harboring either the R248Q or G245S hotspot mutations and wild type MEFs were a kind gift from Dr. Ute Moll (Stony Brook University) and cultured in DMEM with 10% FBS.

### RNA isolation, quantitative RT-PCR and RT^2^ Profiler™ PCR array

RNA extraction and cDNA synthesis were performed using PureLink® RNA Mini Kit and SuperScript III First-Strand Synthesis kit (Life technologies) respectively and according to the manufacturer's protocol. The cDNA was then diluted (1:15) in RNAse-free water, and 5 μl was used in a total reaction volume of 20 μl. Each 20-μl real-time PCR contained a ratio of 10:1:4 (iTaq: Taqman probe (20X): nuclease-free water). PCR was carried out using the Applied Biosystems 7500 Real-Time PCR System (Applied Biosystems, Foster City, CA, USA). The following Taqman probes (life technologies) were used: human *BMP4* (ID: Hs03676628_s1), human *CHK1* (ID: Hs00967506_m1), human *IL6* (ID: Hs00985639_m1), human *IL8* (ID: Hs00174103_m1), human *CXCL1* (ID: Hs00236937_m1), *CXCL10* (ID: Hs01124251_g1), *TGF-B* (ID: Hs00820148_g1), *VEFGA* (ID: Hs00900055_m1) and human *β-actin* (ID: Hs99999903_m1) that was used as a housekeeping gene. Cycle threshold (Ct) values were obtained for each gene of interest and β-actin. ΔCt values were calculated and the relative gene expression normalized to control samples was calculated from ΔΔ Ct values. RT^2^ Profiler™ PCR human Cytokines & Chemokines array from Qiagen (Valencia, CA) was performed according to the manufacturer's. Data was then analyzed using the Excel-based data analysis template provided by Qiagen. Data analysis is based on the ΔΔC_T_ method with normalization of the raw data to either housekeeping genes.

### Western blot analysis

Cultured or transfected cells were washed with ice cold PBS and then directly lysed in cold RIPA buffer containing 1 mM sodium orthovanadate, 2 mM PMSF, and protease inhibitor cocktail (Santa Cruz Biotechnology). Cellular lysates were then clarified by centrifugation at 14,000 rpm for 10 min at 4°C; protein concentration was quantitated by BCA Protein Assay kit from Thermo Scientific (Suwanee, GA). Equal amounts of protein (25 μg) were boiled in Laemmli buffer (Boston Bio Product), and separated on SDS-PAGE (4-15%, Tris-HCl) using the Bio-Rad Criterion system. Separated proteins were then transferred onto nitrocellulose membranes (Bio-rad) and blocked with 5% non-fat milk in PBS-0.1% Tween-20 (PBS-T) for at 1 hour at RT. Primary antibodies diluted 1:1000 or 1:20000 for β-actin were then added to membranes and incubated at 4°C overnight. Membranes were washed 3 times with PBS-T then incubated with diluted 1:5000 HRP-conjugated secondary antibodies for 1 hour at room temperature. Membranes were then incubated with Pierce ECL Western Blotting Substrate (Pierce) and exposed to X-ray films that were then processed and scanned. Anti-total CHK1, anti-Phospho[296]-CHK1, anti-Phospho[317]-CHK1, anti-Phospho[345]-CHK1, anti-p53, anti-caspase 2, anti-Phospho-ATM, anti-IKB-alpha, anti-β-actin, anti-GAPDH and anti-p21 were from Cell Singaling Technology (Danvers, MA). RIPA lysis buffer system, HRP-labeled secondary antibodies, anti-PIDD, anti-Arf6 and anti-PARP were from Santa Cruz Biotechnology (Santa Cruz, CA).

### Bimolecular fluorescence complementation

As described previously [32, 33]. The plasmids pBIFC-C2-CARD VC and pBIFC-C2-CARD VN were kindly provided by Dr. Douglas Green (St. Jude's Children Hospital). pDsRed-Mito purchased from Clonetech (Mountain View, CA).

### Measurement of cytokine levels in media

The ELISA kit for human BMP4 and IL6 were obtained from R&D Systems (Minneapolis, MN). Measurement of secreted cytokine in DMEM was done according to the manufacturer's protocol. Secreted protein levels were normalized to the total amount of cellular protein determined by BCA assay.

### Luciferase assay

As described in [34].

### Microvesicle visualization and isolation

Live cell imaging analysis was performed on a Leica TCS SP8 scanning-laser confocal microscope in a chamber at 37°C and 5% CO_2_. Briefly, MDA-MB-231 cells were grown on poly-D-lysine-coated 35-mm confocal dishes (MatTek Corporation). The following day, media was changed and the cells were treated with 0.8uM Doxorubicin for 24hours. Thirty minutes prior to imaging, Alexa Fluor® 488 Annexin-V and PI (at concentrations indicated in manufacture's protocol) were added to the media to label the membrane and nucleic acids of the microvesicles, respectively. For TMV isolation MDA-MB-231 cells were grown in DMEM supplemented with particle-free FBS for 68 hours. Media was then subjected differential centrifugation as follows: 1000 rpm for 15 minutes, 10,000 rpm for 30 minutes and 50,000xg for 2 hours. The 50,000xg was then washed with PBS and spun for an additional hour at 50,000xg.

### Statistical analysis

The data are represented as the means ± S.E. Unpaired Student's *t* test and two-way ANOVA with Bonferroni post-test statistical analyses were performed using Prism/GraphPad software.

## SUPPLEMENTARY FIGURES




