# Supplementary material for: CHK1 regulates NF-κB signaling upon DNA damage in p53- deficient cells and associated tumor-derived microvesicles
Source: Oncotarget. 2016 Feb 22;7(14):18159–70. doi: 10.18632/oncotarget.7566 (PMC4951279; doi:10.18632/oncotarget.7566)
Supplement: Supplementary file 1 [file oncotarget-07-18159-s001.pdf]

SUPPLEMENTARY FIGURES

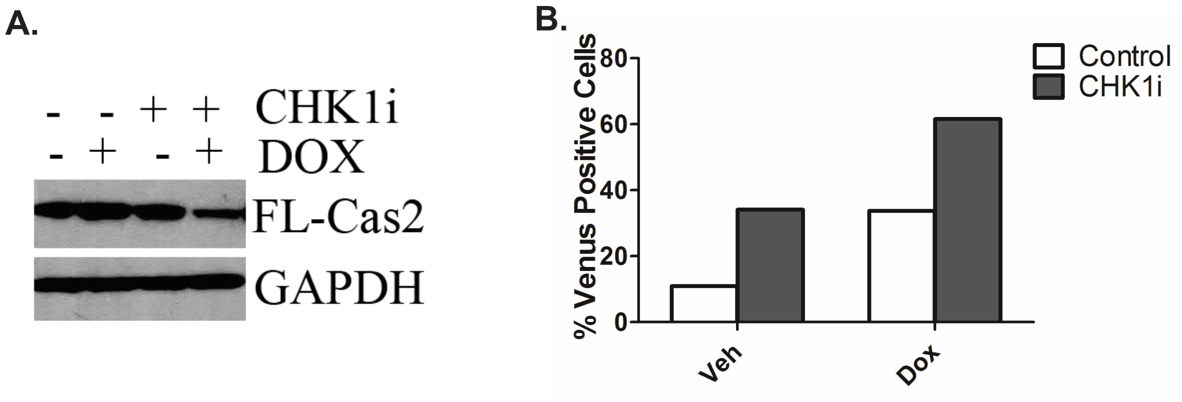

Supplementary Figure S1: CHK1 mediates Caspase 2 activation in a p53-dependent manner. A. MCF7 cells were pretreated for 1 hour with 0.3uM CHK1 inhibitor (AZD7762) or DMSO followed by treatment with 0.4uM Dox or vehicle for 24 hours. Cells were then harvested in RIPA buffer and total cell lysate was analyzed by western blot for the proteins indicated. **B.** MCF7 cells were transiently transfected with C2-CARD VN (500ng) and C2-CARD VC (500ng) along with pshooter.dsRed-mito (250ng) as a reporter for transfection. Twenty-four hours after transfection, cells were pretreated for 1 hour with 0.3uM CHK1 inhibitor (AZD7762) or DMSO followed by treatment with 0.4uM Dox or vehicle for 24 hours. Then the percentage of pshooter.ds.Red-mito-positive (red) cells that were Venus positive (green) was determined from a minimum of 100 cells per plate. in RIPA buffer and total cell lysate was analyzed by western blot for the proteins indicated.

A.

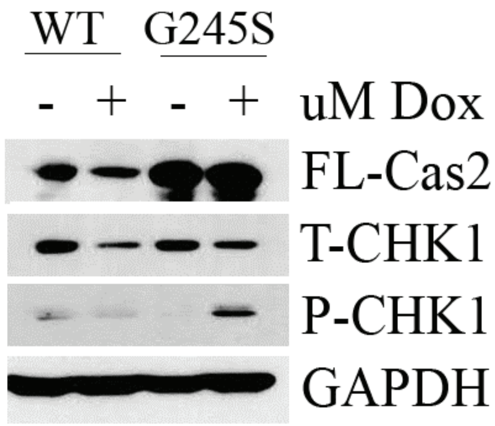

Supplementary Figure S2: P53 deficiency triggers deregulation of the CHK1-caspase-2 pathway. WT or HUPK1 G245S MEFs were treated with 0.2uM Dox for 24h, harvested in RIPA buffer and total cell lysate was analyzed by western blot for the proteins indicated.

A.

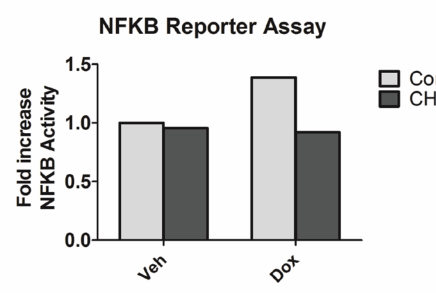

B.

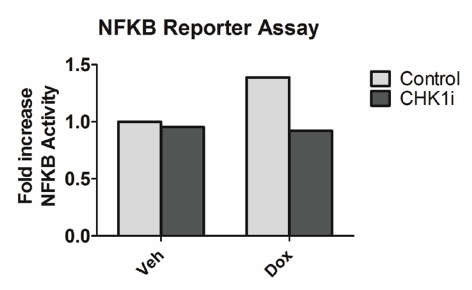

C.

Gene	Fold Regulation	Gene	Fold Regulation	Gene	Fold Regulation	Gene	Fold Regulation	Gene	Fold Regulation	Gene	Fold Regulation
ADIPOQ	-1.9373	CCL24	-1.9381	CXCL16	-1.9373	IL17F	-3.86	IL8	-1.9427	TNFSF11	-1.9589
BMP2	-1.9942	CCL3	-1.9621	CXCL2	-1.9942	IL18	-1.9427	IL9	-1.9641	TNFSF13B	-1.9595
BMP4	-7.9218	CCL5	-4.0086	CXCL5	-7.9218	IL1A	-1.9641	LIF	-3.8498	VEGFA	-3.877
BMP6	-3.8613	CCL7	1.0194	CXCL9	-3.8613	IL1B	-3.8498	LTA	-1.934	XCL1	-1.9328
BMP7	-2.0126	CCL8	-1.952	FASLG	-2.0126	IL1RN	-1.934	LTB	1.0365	ACTB	1
C5	-1.9802	CD40LG	-1.9593	GPI	-1.9802	IL2	1.0365	MIF	1.0062	B2M	-1.9466
CCL1	1.0246	CNTF	-1.9461	IFNA2	1.0246	IL21	1.0062	MSTN	1.0494	GAPDH	-3.9306
CCL11	-1.9665	CSF1	-3.9428	IFNG	-1.9665	IL22	1.0494	NODAL	-1.9856	HPRT1	-3.8914
CCL13	1.0511	CSF2	-1.9795	IL10	1.0511	IL23A	-1.9856	OSM	-1.9564	RPLP0	-1.9516
CCL17	-1.9712	CSF3	-1.0096	IL11	-1.9712	IL24	-1.9564	PPBP	-1.9493	HGDC	1.0426
CCL18	-1.9603	CX3CL1	-1.9901	IL12A	-1.9603	IL27	-1.9493	SPP1	1.0331	RTC	1.0439
CCL19	1.0282	CXCL1	-1.9599	IL12B	1.0282	IL3	1.0331	TGFB2	-1.9256	RTC	-1.9261
CCL2	-1.945	CXCL10	-3.8706	IL13	-1.945	IL4	-1.9256	THPO	-1.9564	RTC	-1.9539
CCL20	-1.9324	CXCL11	-2.0054	IL15	-1.9324	IL5	-1.9564	TNF	-3.9446	PPC	-1.9616
CCL21	-1.9648	CXCL12	-1.9572	IL16	-1.9648	IL6	-3.9446	TNFRSF11B	-1.9593	PPC	-1.948
CCL22	-1.9616	CXCL13	1.0198	IL17A	-1.9616	IL7	-1.9593	TNFSF10	-1.9657	PPC	-1.9539

D.

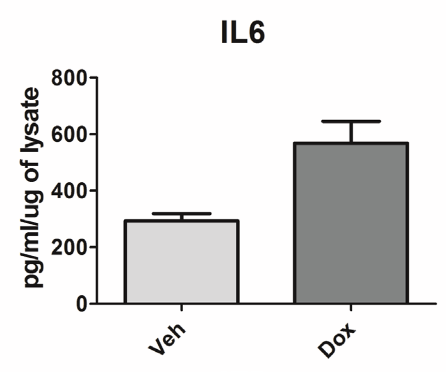

Supplementary Figure S3: CHK1 levels regulate NF-κB signaling in p53-deficient cells in response to doxorubicin. **A.** WT and HUPK1 G245S MEFs were co-transfected with 1ug of NFKB promoter-luciferase construct and V5 luciferase construct for 18 hours. MEFs were then pretreated for 1 hour with 0.3uM CHK1 inhibitor (AZD7762) or DMSO followed by treatment with 0.2uM doxorubicin for 24 hours. Luciferase and galactosidase activities were extracted and assayed as described in "Material and Methods" and measured luciferase activity was normalized to measured galactosidase activity. **B.** MCF7 cells were co-transfected with 1ug of NFKB promoter-luciferase construct and V5 luciferase construct for 18 hours, followed by treatment with 0.8uM doxorubicin for 24 hours. Luciferase and galactosidase activities were extracted and assayed as described in "Material and Methods" and measured luciferase activity was normalized to measured galactosidase activity. **C.** MDA-MB-231 cells were transfected with CHK1 siRNA (20nM). Forty-eight hours after transfection, cells were treated with 0.8uM doxorubicin for 24 hours. RNA was then extracted, cDNA generated and a RT^2 Profiler^{TM} PCR human Cytokines & Chemokines array was used to assess mRNA levels. Data was then analyzed using the Excel-based data analysis template provided by Qiagen. Data analysis is based on the $\Delta\Delta C_T$ method with normalization of the raw data to either housekeeping genes. **D.** MDA-MB-231 cells were treated with 0.8uM doxorubicin for 24 hours, media was then collected and ELISA was performed to assess levels of IL6 protein in the media. Data are presented as mean ± SEM of 2 independent experiments.

A.

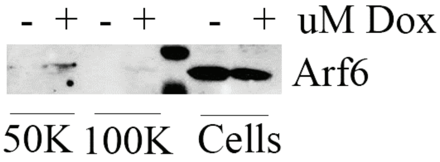

Supplementary Figure S4: Doxorubicin induces the shedding of TMVs containing NF-κB-responsive genes. A. MDA-MB-231 cells were treated 0.8uM doxorubicin for 24 hours. After 24 hours cells were stained with DRAQ5 to label all cells followed by treatment with Annexin-V and Propidium to specifically label. Images were then taken every 10 minutes for 12 hours. **B.** MDA-MB-231 cells were treated 0.8uM doxorubicin for 60 hours. Cells were then harvested in RIPA buffer and media was then collected and subjected to differential centrifugation (outlined in “material and methods”). Protein was then extracted from the 50,000xg, 100,000xg pellet and cells. Lysate was analyzed by western blot for the protein indicated.

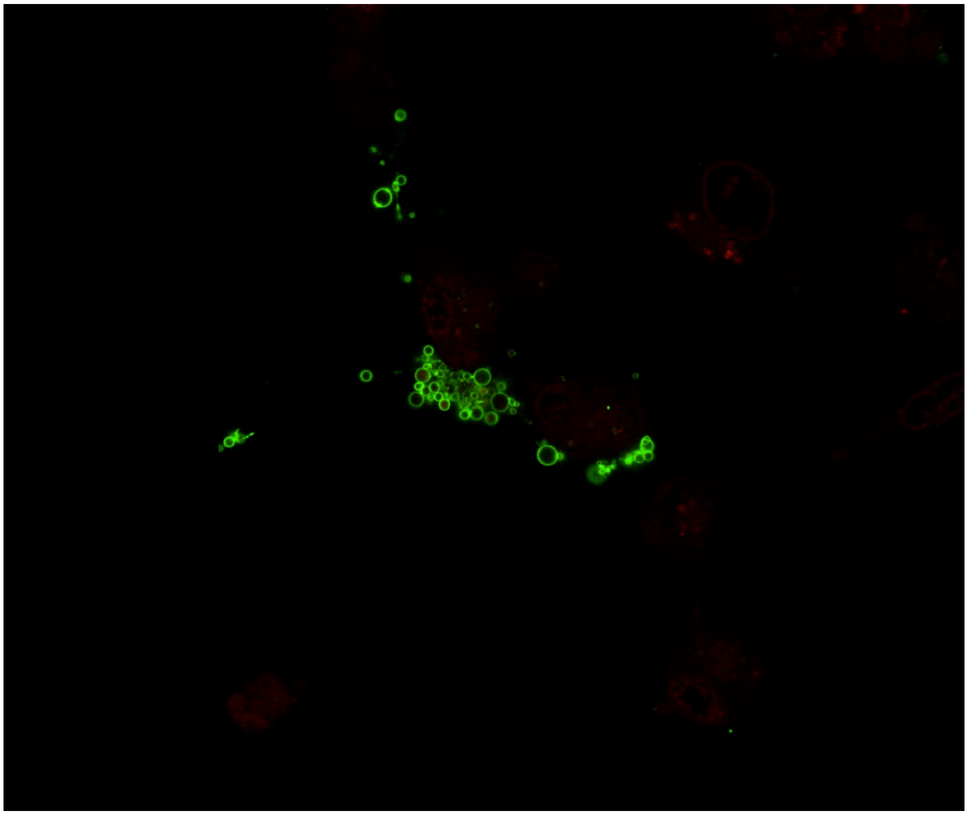

See Supplementary File 1
